# Supplementary material for: Improved Understanding of Interactions between Risk Factors for Child Obesity May Lead to Better Designed Prevention Policies and Programs in Indonesia
Source: Nutrients. 2020 Jan 8;12(1):175. doi: 10.3390/nu12010175 (PMC7019618; doi:10.3390/nu12010175)
Supplement: Supplementary file 1 [file nutrients-12-00175-s001.pdf]

Table S1. SEMI QUANTITATIVE FOOD FREQUENCY (SQFF) FORM

Guideline:

- Write down the frequency of consumption of each type of food / food ingredients in days, weeks, 1 month, 3 months or never consume
- Household portion: plate, spoon, slice, glass, bowl, etc.

A. Junk Food

No.	Brand name	Amount of consumption (each time)		Frequency of consumption				Energy (Kcal)	Saturated fat (grams)	Carb (grams)	Natrium (grams)
		Household portion	Grams	Day	Week	1 month	3 month				
A.	**Sweet cereals**										
	Instant cereal (Energen®)										
	Instant cereal (Koko Crunch®)										
	Others, specify….										
B.	**Instant rice / noodle products**										
	Instant noodles (Migelas®)										
	Fried noodles ready to eat (Spix Mie®)										
	Instant noodles (Indomie®)										
	Instant porridge (Super Bubur®)										
	Others, specify….										
C.	**Sweet snacks**										
	Richeese® wafer										
	Tango® wafer										
	Tango® waffle										
	Goriorio® biscuit										
	Gery Toya-toya® biscuit										
	Bimbim® biscuit										
	Selamat ® biscuit										
	Oreo® biscuit										
	Others, specify….										
D.	**Processed Meat**										
	Chicken sausage (So Nice®)										
	Fish Sausage										
	Beef sausage										
	Goat Sausage										
	Others, specify….										
**E.**	**Salty Snacks**										
	Potato chips (Chitato ®)										
	Potato chips (Leo®)										
	Pilus Garuda										
	cheese wafer (Zuper®)										
	Pop Corn										
	Cracker										
	Salted Beans										
	Others, specify….										
F.	**Commercial Juices/ Fruit Drinks**										
	Buavita®										
	Minute Maid Pulpy Orange®										
	Tipco Kalbe®										
	Others, specify….										
G.	**Milk and milk products**										
	ULTRA milk®										
	Frisian Flag®										
	Dancow®										
	MILO®										
	Sweetened condensed milk										
	Cheese (KRAFT®)										
	Yoghurt										
	Others, specify….										

**H.**	**Ice cream**										
	• Walls®										
	• Magnum®										
	• Paddle Pop®										
	Others, specify….										
	**Campina**										
	• Bazooka®										
	• Concerto®										
	Others, specify….										
I.	**Chocolate and Candy**										
	Candy (Relaxa®)										
	Candy (Kiss®)										
	Candy (Milkita®)										
	Chocolate (SilverQueen®)										
	Chocolate (Cadburry®)										
	Others, specify….										
**J.**	**Western Fast Food**										
	**Mc Donald’s®**										
	• Beef Burger										
	• Cheese Burger										
	• Double Cheese										
	• Burger Seafood										
	• Big Mac®										
	• Filet o fish®										
	• Mc chicken										
	• Crispy hot chicken										
	• Soft Drinks										
	• Ice Cream										
	Others, specify….										
	**Kentucky Fried Chicken®**										
	• Original fried chicken										
	• Extra crispy fried chicken										
	• Kentucky nuggets										
	• Kentucky fried										
	• Spaghetti										
	• Corncake (Perkedel)										
	• French fries										
	• Corn soup										
	• Cream soup										
	• Ice cream										
	Others, specify….										
	**Dunkin Donuts®**										
	• Big donuts										
	• Mini donuts										
	• Fruits Juice										
	• Ice cream										
	Others, specify….										
	**Pizza Hut®**										
	• Supreme										
	• Black paper chick										
	• Meat Lover										
	• American Favorite										
	• Favorite Beef										
	• Thousand Island										
	• Chicken Favorite										
	• Spaghetti										
	• Garlic Bread										
	• Salad										
	• Milk Shake										
	• Ice Cream										
	Others, specify….										
	**Local Fast Food**										
	Meatball										
	Fried meatball and tofu (Batagor)										
	Others, specify….										

**K.**	**Frozen Food**										
	Chicken Nugget Fiesta®										
	Dumplings (Siomay)										
	Others, specify….										
**L.**	**Fat and fat -based products**										
	Butter										
	Margarine										
	Homemade butter										
	Cream soup										
	Others, specify….										
**M.**	**Sugar Sweetened Beverages (SSB)**										
	**Carbonated**										
	Coolant®										
	Coca cola®										
	Fanta®										
	Sprite®										
	Big Cola®										
	Others, specify….										
	**Noncarbonated**										
	Segar Sari Frenta®										
	Frutkuat®										
	Tea Rio®										
	Tea Pucuk Harum®										
	Tea Teh gelas®										
	Tea Frutz®										
	Tea Sisri®										
	Tea Mount Tea®										
	Ale-ale®										
	Nutrisari®										
	Syrup										
	Mizone®										
	Pocari Sweat®										
	Others, specify….										
**N.**	**Others**										
	**Food flavoring(MSG)**										
	• Royco®										
	• Masako®										
	• Others, specify….										
	**Supplement**										
	• Sakatonik ABC®										
	• Others, specify….										

Non-Junk Food

**No.**	**Brand name**	**Amount of consumption (each time)**		**Frequency of consumption**				Energy (Kcal)	Saturated fat (grams)	Carb (gram)	Natrium (gram)
		Household portion	Gram	Day	Week	1 month	3 month				
**1.**	**Staple food**										
	- rice										
	- flour										
	- potato										
	- cassava										
	- sweet potato										
	- corn										
	Others, specify….										
**2.**	**Animal-based food**										
	- Beef										
	- Lamb										
	- Broiler chicken										
	- Local chicken										
	- Broiler chicken eggs										
	- Local chicken eggs										
	- Quail eggs										
	- Duck eggs										
	- Goose eggs										
	- Beef liver										
	- Chicken liver										
	- Gizzard										

	- Pig intestine										
	- Beef intestine										
	- Seawater fish										
	- Freshwater fish										
	Others, specify….										
**3.**	**Plant-based food**										
	- Soybean tempeh										
	- Tofu										
	- Tauco										
	Others, specify….										
4.	**Vegetables**										
	Spinach										
	Bean										
	Broccoli										
	Cauliflower										
	Red chili pepper										
	Big green chili										
	Cayenne pepper										
	Leek										
	Cassava leaves										
	Papaya leaf										
	Lettuce										
	Celery leaves										
	Basil leaves										
	Melinjo Leaves										
	White Mustard Leaves										
	Green Mustard Leaves										
	Sweet potato leaves										
	Long Bean Leaves										
	Taro Leaves										
	Luffa										
	Yellow velvetleaf										
	Young jackfruit										
	Djenkol (Jengkol)										
	Young corn										
	Banana Heart										
	Fresh mushrooms										
	Kale										
	Cabbage / Cabbage										
	Breadnut										
	Cosmos leaf										
	Long beans										
	Pea										
	Cucumber										
	Chayote										
	Pumpkin (Waluh)										
	Bitter melon										
	Young papaya										
	Bamboo shoots										
	Breadfruit										
	Sprouts / sprouts										
	Eggplant										
	Tomato										
	Carrot										
	Others, specify….										
**5.**	**Fruit**										
	Avocado										
	Grape										
	Apple										
	Star fruit										
	Jicama										
	Java plum										
	Langsat (Duku)										
	Durian										
	Longan										
	Kedongdong (Ambarella)										
	Small cherry										
	Wax apple										
	Guava										
	Cashew fruit										
	Pomelo										
	Sweet orange										
	Mango (Harum manis)										
	Mango (Golek)										

	Mango (Kweni)										
	Pometia pinnata										
	Passion fruit										
	Mangosteen										
	Melon										
	Dragon fruit										
	Jackfruit Cook										
	Pineapple										
	Papaya (mature)										
	Banana (Ambon)										
	Banana (Plantain)										
	Banana (Pisang susu)										
	Banana (Pisang kapok)										
	Pear										
	Rambutan										
	Sapodilla (Sawo)										
	Watermelon										
	Snakefruit (Salak)										
	Soursop										
	Sugar-apple										
	Strawberry										
	Kiwi										
	Others, specify….										
**6.**	**Oil-fat**										
	Palm oil										
	Corn oil										
	Chicken Fat										
	Others, specify….										
7.	**Beverages**										
	**a. Tea**										
	- Sweet tea										
	- Non sugary tea										
	Others, specify….										
	**b. Fruit Juice**										
	- Guava juice										
	- Mango juice										
	- Avocado juice										
	Others, specify….										

Table S2. MODIFIED CHILDREN PHYSICAL ACTIVITY QUESTIONNAIRE (M-CPAQ)

Note:

- Column 1 shows period information
- Column 2 shows specific activities
- Column 3 shows what time the specific activities done
- Column 4 indicates whether the specific activities done or not
- Column 5 shows frequency of specific activities
- Column 6 shows duration (in minute/hour) of specific activities done
- Column 7 shows what TV program watched. Only for **screen-based activities**

1 Period	2 Specific activities	A. Monday – Friday					B. Saturday				
		3A Time	4A Yes / No	5A How many times?	6A Duration (minute/ hour)	7A TV program	3A Time	4A Yes / No	5A How many times?	6A Duration (minute/ hour)	7A TV program
Wake up to go to school	What time do you wake up in the morning? ………….. am										
	Screen-based activity										
	Watching TV		Yes No					Yes No			
	Playing (PlayStation, Gameboy, etc.)		Yes No					Yes No			
	Using computer/laptop/tablet		Yes No					Yes No			
	Others, specify ___________		Yes No					Yes No			
	**Physical activity**										
	Cleaning up the rooms / house (e.g. sweeping)		Yes No					Yes No			
	Washing (clothes, plates)		Yes No					Yes No			
	Clean yourself (showering, clothes)		Yes No					Yes No			
	Breakfast		Yes No					Yes No			
	Others, specify ___________		Yes No					Yes No			
	What time do you go to school? ……….. am										
At school	Study		Yes No					Yes No			
	Study physical activity education (PE) Specify____________		Yes No					Yes No			
	Study computer		Yes No					Yes No			
	Commute to school using car (round trip)		Yes No					Yes No			
	Commute to school using bike (round trip)		Yes No					Yes No			
	Commute to school by walk (round trip)		Yes No					Yes No			
	During recess<br>- Sit and chat<br>- Study/ do assignment<br>- Play______<br>Eat/ snack		 Yes No<br>Yes No<br>Yes No<br>Yes No					 Yes No<br>Yes No<br>Yes No<br>Yes No			
	Extracurricular<br>- Scout<br>- Computer<br>- Dance		 Yes No<br>Yes No<br>Yes No					 Yes No<br>Yes No<br>Yes No			

1<br>Period	2<br>Specific activities	A. Monday – Friday					B. Saturday				
		3A<br>Time	4A<br>Yes / No	5A<br>How many times?	6A<br>Duration (minute/ hour)	7A<br>TV program	3A<br>Time	4A<br>Yes / No	5A<br>How many times?	6A<br>Duration (minute/ hour)	7A<br>TV program
	- Marching band<br>Others, specify________		Yes No<br>Yes No					Yes No<br>Yes No			
	Additional lessons		Yes No					Yes No			
	Others, specify __________		Yes No					Yes No			
After school until sunset	***Screen-based activity***										
	Watching TV		Yes No					Yes No			
	Playing (PlayStation, Gameboy, etc.)		Yes No					Yes No			
	Using computer/laptop/tablet		Yes No					Yes No			
	Others, specify __________		Yes No					Yes No			
	**Physical activity**										
	Lunch		Yes No					Yes No			
	Cleaning up the rooms / house (e.g. sweeping)		Yes No					Yes No			
	Washing (clothes, plates)		Yes No					Yes No			
	Clean yourself (showering, clothes)		Yes No					Yes No			
	Siestas		Yes No					Yes No			
	Qur’an class/ course		Yes No					Yes No			
	Play<br>- Football<br>- Bike<br>- Badminton<br>- Skip rope<br>- Playing cards<br>- Board game (chees, monopoly, etc.)<br>Others______		 Yes No<br>Yes No<br>Yes No<br>Yes No<br>Yes No<br>Yes No<br>Yes No					 Yes No<br>Yes No<br>Yes No<br>Yes No<br>Yes No<br>Yes No<br>Yes No			
	Others, specify___________		Yes No					Yes No			
At bedtime	***Screen-based activity***										
	Watching TV		Yes No					Yes No			
	Playing (PlayStation, Gameboy, etc.)		Yes No					Yes No			
	Using computer/laptop/tablet		Yes No					Yes No			
	Others, specify __________		Yes No					Yes No			
	**Physical activity**										
	Study/ do assignment		Yes No					Yes No			
	Dinner		Yes No					Yes No			
	Read (novel, magazine, comic, etc.)		Yes No					Yes No			
	Others, specify___________		Yes No					Yes No			
	What time do you sleep at night? …….. pm										

SUNDAY

1. Period	2.Time	3. Specific activities	4.Yes No	5. How many times	6. Duration (minute/ hour)	7. TV Program
Morning to afternoon		What time do you wake up in the morning? ….. am				
		Screen-based activity				
		Watching TV	Yes No			
		Playing (PlayStation, Gameboy, etc.)	Yes No			
		Using computer/laptop/tablet	Yes No			
		Others, specify __________	Yes No			
		**Physical activity**				
		Cleaning up the rooms / house (e.g. sweeping)	Yes No			
		Washing (clothes, plates)	Yes No			
		Clean yourself (showering, clothes)	Yes No			
		Breakfast	Yes No			
		Qur'an class/ course	Yes No			
		Play<br>- Football<br>- Bike<br>- Badminton<br>- Rope skip<br>- Playing card<br>- Board game (chees, monopoly, etc.)<br>Others______	 Yes No<br>Yes No<br>Yes No<br>Yes No<br>Yes No<br>Yes No<br>Yes No			
		Others, specify_____________________	Yes No			
After school until sunset		***Screen-based activity***				
		Watching TV	Yes No			
		Playing (PlayStation, Gameboy, etc.)	Yes No			
		Using computer/laptop/tablet	Yes No			
		Others, specify __________	Yes No			
		**Physical activity**				
		Siesta	Yes No			
		Traveling with family	Yes No			
		Qur'an class/ course	Yes No			
		Play<br>- Football<br>- Bike<br>- Badminton<br>- Rope skip<br>- Playing card<br>- Board game (chees, monopoly, etc.)<br>Others ______	 Yes No<br>Yes No<br>Yes No<br>Yes No<br>Yes No<br>Yes No<br>Yes No			
		Afternoon bath	Yes No			
		Others, specify_____________________	Yes No			
At bedtime		***Screen-based activity***				
		Watching TV	Yes No			
		Playing (PlayStation, Gameboy, etc.)	Yes No			
		Using computer/laptop/tablet	Yes No			
		Others, specify __________	Yes No			
		**Physical activity**				
		Study/ do assignment	Yes No			
		Dinner	Yes No			
		Read (novel, magazine, comic)	Yes No			
		Others, specify __________	Yes No			
		What time do you sleep in the night? ……….pm				
